# Variceal Upper Gastrointestinal Bleeding: A Retrospective Cohort of 98 Cases, Historical Comparison, and Updated Management Algorithm

**DOI:** 10.3390/life15101626

**Published:** 2025-10-17

**Authors:** Laurențiu Augustus Barbu, Liliana Cercelaru, Ionică-Daniel Vîlcea, Valeriu Șurlin, Stelian-Stefaniță Mogoantă, Tiberiu Stefăniță Țenea Cojan, Nicolae-Dragoș Mărgăritescu, Ana-Maria Țenea Cojan, Valentina Căluianu, Daniela Marinescu, Gabriel Florin Răzvan Mogoș, Liviu Vasile

**Affiliations:** 1 Department of Surgery, Railway Clinical Hospital Craiova, University of Medicine and Pharmacy of Craiova, 2 Petru Rares Street, 200349 Craiova, Romania; laurentiu.barbu@umfcv.ro (L.A.B.); tiberiu.tenea@umfcv.ro (T.S.Ț.C.); valentina.andronache@yahoo.com (V.C.); gabriel.mogos@umfcv.ro (G.F.R.M.); 2 Department of Embryology and Anatomy, University of Medicine and Pharmacy of Craiova, 200349 Craiova, Romania; liliana.cercelaru@umfcv.ro; 3 Department of Surgery, Emergency County Hospital, University of Medicine and Pharmacy of Craiova, 2 Petru Rares Street, 200349 Craiova, Romania; ionica.vilcea@umfcv.ro (I.-D.V.); vsurlin@gmail.com (V.Ș.); ssmogo@yahoo.com (S.-S.M.); dtmarinescu@yahoo.com (D.M.); vliviu777@yahoo.com (L.V.); 4 Faculty of Medicine, University of Medicine and Pharmacy of Craiova, 200349 Craiova, Romania; anamariatenea0324@gmail.com

**Keywords:** variceal upper gastrointestinal bleeding, cirrhosis, admission delay, hemoglobin, Kaplan–Meier, restrictive transfusion, endoscopic band ligation, pre-emptive TIPS, portal hypertension, prognosis

## Abstract

**Background**: Variceal upper gastrointestinal bleeding (VUGIB) remains a major cause of short-term mortality in cirrhosis despite advances in endoscopic and pharmacological therapy. Prognostic factors and outcomes were evaluated in a historical cohort, and a guideline-aligned management algorithm is proposed. **Methods**: We conducted a retrospective study of 98 consecutive adults admitted with VUGIB to a tertiary surgical center in Romania (2009–2014). Demographics, etiology, admission hemoglobin (Hb), timing of presentation, endoscopic and surgical management, and outcomes were recorded. Survival was analyzed using Kaplan–Meier with log-rank tests; associations were tested with chi-square and *t*-tests; predictors of mortality were assessed with logistic regression. Receiver operating characteristic (ROC) curve analysis was used to evaluate the discriminatory ability of hemoglobin for in-hospital mortality. **Results**: Mean age was 57.8 ± 11.7 years; 60.2% were male. Cirrhosis etiology was alcoholic in 73%, viral in 18%, and metabolic in 9%. Endoscopy occurred within 48 h in 62% of patients, but only 4% underwent the procedure within 8 h. Overall mortality was 17.3%. Kaplan–Meier analysis showed no survival difference between alcoholic and viral cirrhosis (log-rank *p* = 0.39), but survival was markedly lower with Hb < 8 g/dL (*p* < 0.001). Admission delay was prognostic: >24 h was associated with worse survival (*p* < 0.05). On multivariable analysis (reference 1–2 days), admission at 3–4 days (OR 35.3, 95% CI 1.6–786, *p* = 0.024), >4 days (OR 71.0, 95% CI 2.16–2337, *p* = 0.017), and <6 h (OR 22.4, 95% CI 1.25–399.7, *p* = 0.035) independently predicted death. Admission Hb predicted mortality with an AUC of 0.79; the optimal cut-off was 4.3 g/dL (sensitivity 57%, specificity 95%). Limited use of pre-emptive TIPS likely contributed to outcomes. **Conclusions**: In this historical cohort, mortality from VUGIB was driven mainly by bleeding severity and admission delay, rather than by cirrhosis etiology. The study provides a historical benchmark for Eastern Europe, highlights gaps in adherence to contemporary standards, and supports restrictive transfusion, early vasoactive therapy, antibiotics, urgent endoscopy, and pre-emptive TIPS. The retrospective single-center design and limited therapies available during the study period remain important limitations.

## 1. Introduction

Upper gastrointestinal bleeding (UGIB) remains one of the most frequent and life-threatening emergencies encountered in gastroenterology and surgery. Despite significant advances in diagnosis and treatment, UGIB continues to be associated with considerable morbidity and mortality, with reported rates ranging from 7% to 10% in large international cohorts [1]. In Romania and many Eastern European countries, the burden of UGIB is particularly high due to the prevalence of alcohol-related liver disease and viral hepatitis, both of which are predisposed to variceal hemorrhage [2].

Variceal bleeding is the most severe form of UGIB, typically occurring in patients with advanced cirrhosis and portal hypertension. Approximately 50% of cirrhotic patients develop esophageal varices during the course of their disease, with a third of these experiencing at least one episode of variceal bleeding [3]. Mortality during the first episode remains high, ranging from 15% to 25%, largely due to hemodynamic instability, comorbidities, and the risk of early rebleeding [4]. Several prognostic factors have been identified, including advanced age, low hemoglobin at admission, hemodynamic shock, and delayed access to hospital care [5].

Current international guidelines emphasize that patients with suspected variceal upper gastrointestinal bleeding should receive vasoactive drugs at presentation, be managed with a restrictive transfusion policy, and undergo endoscopic treatment within 12 h after stabilization [6]. Nevertheless, observational studies continue to show delays in hospital referral and heterogeneity in treatment approaches, factors that may adversely influence prognosis [7].

The objective of this study was to identify prognostic factors for in-hospital mortality and to propose a management algorithm aligned with current international guidelines. The analysis focused on demographic and clinical characteristics to explore predictors of in-hospital mortality through multivariate modeling, the prognostic significance of admission hemoglobin using ROC curve analysis, and the interplay between anemia severity and admission delay. In addition, by contrasting our findings with previously published cohorts, we provide a management framework that reflects current international guideline recommendations [6,7].

## 2. Materials and Methods

### 2.1. Study Design and Setting

This retrospective observational cohort included 98 adult patients with variceal upper gastrointestinal bleeding admitted to the First Surgical Clinic of the Emergency County Clinical Hospital of Craiova, Romania, between 1 January 2009 and 31 December 2014.

### 2.2. Inclusion and Exclusion Criteria

Inclusion criteria were patients aged ≥18 years with a confirmed diagnosis of variceal UGIB, identified based on clinical presentation, laboratory data, and upper gastrointestinal endoscopy (UGIE). Exclusion criteria included non-variceal UGIB, lower gastrointestinal bleeding, incomplete medical records, and comorbidities significantly affecting the assessment of bleeding etiology (e.g., malignancies, hematological disorders).

### 2.3. Data Collection

For each patient, the following variables were recorded: demographics (age, sex, and place of residence—rural or urban); etiology of cirrhosis and relevant comorbidities (alcoholic, viral, or metabolic cirrhosis; chronic alcohol use; cardiovascular and gastrointestinal conditions, such as gastritis or peptic ulcer, as well as other significant medical disorders). Risk factors included chronic alcohol consumption, use of nonsteroidal anti-inflammatory drugs (NSAIDs), and anticoagulant therapy. Baseline laboratory parameters collected at admission comprised hemoglobin, hematocrit, platelet count, creatinine, urea, and other relevant values. Timing variables referred to the interval from the onset of bleeding to hospital admission, the time from admission to endoscopy, and the time from admission to surgery when applicable. Endoscopic findings were classified according to the lesions identified during upper gastrointestinal endoscopy. Therapeutic interventions were documented, including medical therapy (e.g., antisecretory drugs), endoscopic procedures (e.g., band ligation), and surgery where necessary. Finally, clinical outcomes were recorded, consisting of mortality, rebleeding episodes, and complications related to the administered therapies.

### 2.4. Endoscopic and Surgical Management

All patients underwent UGIE within 48 h of hospital admission according to ESGE guidelines. Endoscopic interventions included band ligation and sclerotherapy for variceal bleeding. Surgical interventions were performed in cases of failure of endoscopic treatment or recurrent bleeding, with trans-gastric ligation of esophageal varices being the most common surgical procedure.

#### Handling of Missing Data and Endoscopic Protocol

Cases with incomplete medical records were excluded from the analysis to avoid bias from missing data. No imputation methods were applied. All endoscopic procedures were performed according to the institutional protocol in place during 2009–2014, which was aligned with the then-current ESGE recommendations. Endoscopic evaluation and interventions (band ligation or sclerotherapy) were carried out by experienced endoscopists from the same surgical department, following a standardized approach.

### 2.5. Statistical Analysis

Descriptive statistics were used to report demographic and clinical characteristics as means ± standard deviation (SD) for continuous variables and frequencies (percentages) for categorical variables. Survival analysis was performed using Kaplan–Meier curves for categorical variables, and log-rank tests were used to compare survival distributions. Comparative analyses were performed with chi-square tests for categorical data and *t*-tests for continuous data. A *p*-value < 0.05 was considered statistically significant. Data were initially processed using Microsoft Excel 2016 (Microsoft Corp., Redmond, WA, USA) with XLSTAT 2022 (Addinsoft SARL, Paris, France). Statistical analyses were subsequently performed using SPSS version 25.0 (IBM Corp., Armonk, NY, USA).

### 2.6. Ethics Statement

This study was conducted in accordance with the Declaration of Helsinki and was approved by the Ethics Committee of the Emergency County Clinical Hospital (approval number 42408/9 September 2025). Informed consent for the use of medical data for research purposes was obtained in writing from all patients at the time of admission, in full compliance with ethical standards. For patients who subsequently died, informed consent had already been obtained at admission. All data were anonymized prior to analysis. Data analysis and manuscript submission were performed only after receiving this approval.

## 3. Results

The study cohort included 98 patients with variceal upper gastrointestinal bleeding. The majority were male (60.2%), with a mean age of 57.8 ± 11.7 years (Table 1). Most patients originated from rural areas (74.5%). Alcoholic cirrhosis was the leading etiology (73%), followed by viral (18%) and metabolic causes (9%). At admission, nearly half of the patients presented with Grade III bleeding severity (47%), while 16% were classified as Grade IV. Overall mortality was 17.3%.

In most patients, upper endoscopy was performed within 48 h of admission (61 cases, 62%). Only 4 patients (4%) underwent endoscopy within the first 8 h. A considerable proportion experienced delayed procedures: 24 patients (24%) between 3–4 days and 9 patients (9%) after more than 4 days (Table 2).

Endoscopic evaluation confirmed that esophageal varices were the predominant source of upper gastrointestinal bleeding. A total of three patients required surgical intervention, all operated within the first 24 h. These cases underwent trans-gastric ligation of esogastric varices after failure of endoscopic and pharmacological therapy.

All patients received antisecretory medication during hospitalization. Combined therapy with both proton pump inhibitors (PPIs) and H2 receptor antagonists was the most frequently used regimen (51 cases, 52%). Monotherapy with PPIs was administered in 27 patients (28%), while 20 patients (20%) received only H2 receptor inhibitors (Table 3).

The majority of patients presented with a combination of hematemesis and melena (68 cases, 70%). Isolated hematemesis was reported in 18 patients (18%), while melena alone was observed in 12 patients (12%) (Table 4).

At admission, bleeding had already stopped in the majority of patients (60 cases, 61%). Continuous active bleeding was present in 20 patients (20%), while 18 patients (18%) presented with recurrent episodes (Table 5).

**Figure 1:**
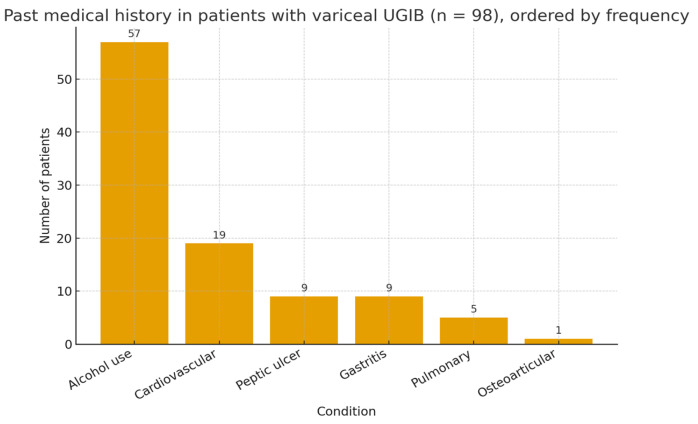
Past medical history in patients with variceal UGIB (n = 98).

Regarding past medical history, the most frequent associated condition was chronic alcohol use, reported in 57% of patients. Cardiovascular comorbidities were found in 19% of cases, while gastrointestinal conditions such as gastritis and peptic ulcer were reported in 9% each. Pulmonary disorders were noted in 5% of patients, and osteoarticular conditions were rare (1%) (Figure 1).

**Figure 2:**
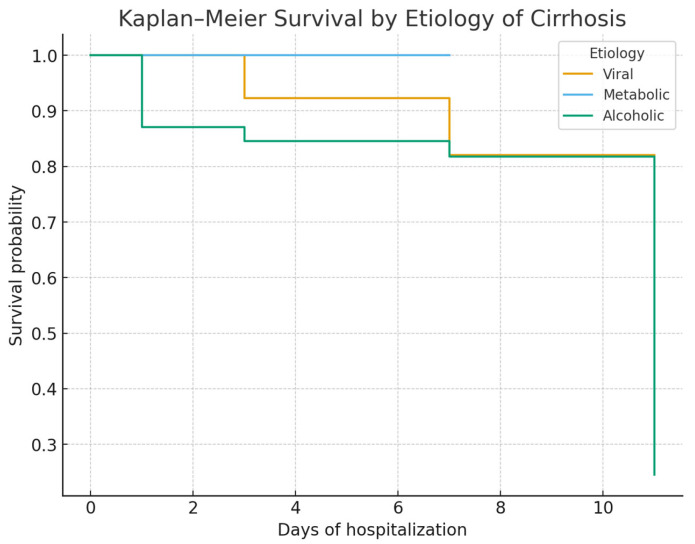
Kaplan–Meier survival curves stratified by the etiology of cirrhosis (alcoholic, viral, metabolic). Survival probability did not differ significantly between alcoholic and viral cirrhosis (log-rank *p* = 0.39). The metabolic subgroup was underrepresented and not suitable for meaningful comparison.

**Figure 3:**
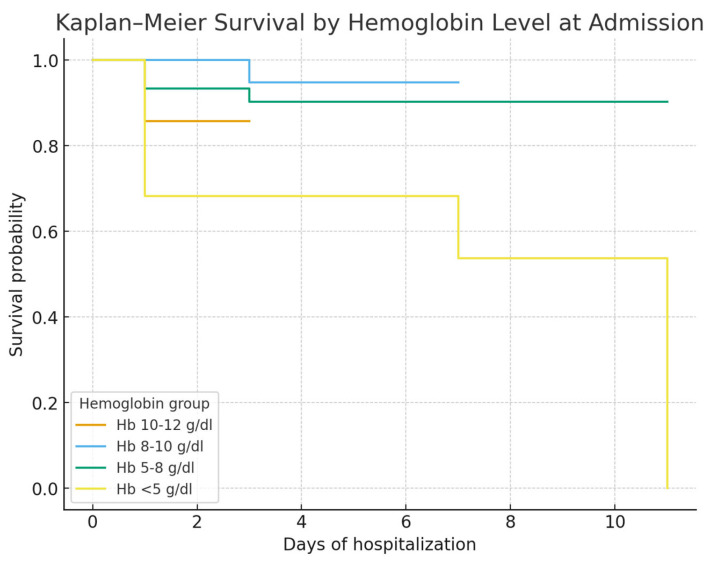
Kaplan–Meier survival by hemoglobin level at admission. Groups: <5 g/dL (n = 16), 5–8 g/dL (n = 46), 8–10 g/dL (n = 26), 10–12 g/dL (n = 10). Survival differed significantly across groups (log-rank *p* < 0.001). Total deaths: 17/98.

**Figure 4:**
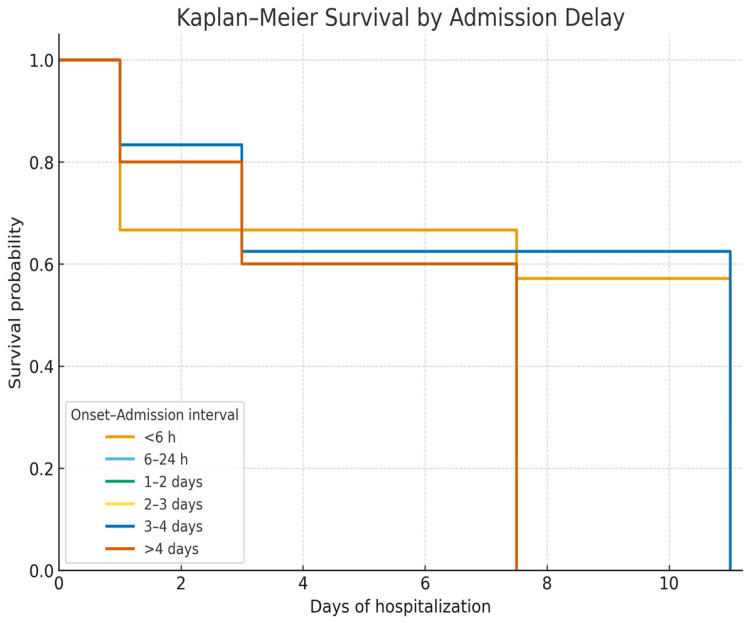
Kaplan–Meier survival curves stratified by admission delay (<6 h, 6–24 h, 1–2 days, 2–3 days, >3 days). Patients admitted more than 24 h after the onset of bleeding had significantly worse survival compared with those admitted earlier (log-rank *p* < 0.05).

Kaplan–Meier survival curves showed no significant survival difference between alcoholic and viral cirrhosis (Figure 2). In contrast, survival was significantly lower in patients admitted with Hb < 8 g/dL (Figure 3) and in those admitted more than 24 h after bleeding onset (Figure 4).

**Figure 5:**
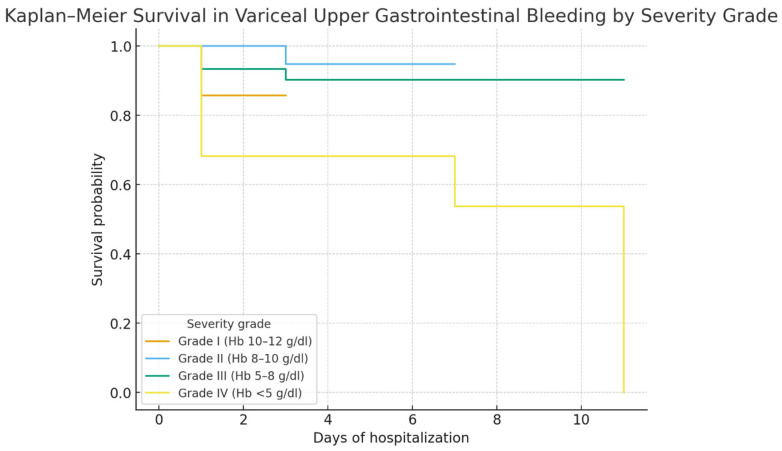
Kaplan–Meier survival curves stratified by severity grade of variceal upper gastrointestinal bleeding (Grade I–IV, defined by hemoglobin level at admission). Survival probability was significantly lower in patients with Grade III (Hb 5–8 g/dL) and Grade IV (Hb < 5 g/dL) compared with those in Grade I−II (Hb ≥ 8 g/dL; log-rank *p* < 0.001).

Kaplan–Meier survival analysis stratified by severity grade (Figure 5) showed a clear prognostic gradient: patients with Grade III (Hb 5–8 g/dL) and Grade IV (Hb < 5 g/dL) had markedly lower survival compared with Grade I–II (Hb ≥ 8 g/dL; log-rank *p* < 0.001). These findings were consistent with Chi-square analysis, which also demonstrated that variceal UGIB grade, admission delay, and length of hospital stay were significantly associated with mortality, while cirrhosis etiology was not.

The final regression model showed a good fit (McFadden’s R^2^ = 0.56; AIC = 65.6; N = 98, deaths = 17). Multivariate logistic regression (Table 6) showed that interval admission delay was a significant predictor of mortality. Compared with patients admitted after 1–2 days, those admitted very early (<6 h) had higher odds of death (OR 22.4, 95% CI 1.25–399.7, *p* = 0.035). Similarly, delayed admission of 3–4 days (OR 35.3, 95% CI 1.59–786.1, *p* = 0.024) or more than 4 days (OR 71.0, 95% CI 2.16–2337.1, *p* = 0.017) was strongly associated with increased mortality. In contrast, etiology, age group, and variceal bleeding grade were not significantly associated with outcome.

In the univariate analysis, admission delay ≥3 days was likewise significantly associated with mortality (OR 6.82, 95% CI 1.87–24.9, *p* = 0.004), while admission delay >24 h alone was not (OR 0.88, *p* = 0.805) (Table 7).

Mortality was highest in patients with Hb < 5 g/dL and in those admitted after >4 days or within 6 h of bleeding onset (Table 8).

**Figure 6:**
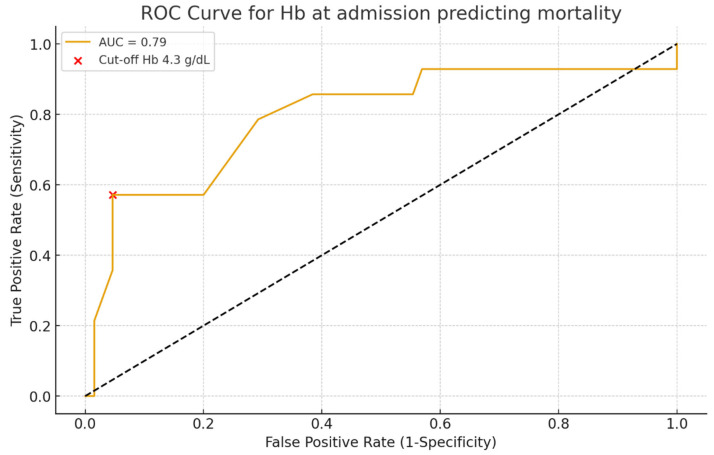
ROC curve of admission hemoglobin predicting in-hospital mortality (AUC = 0.79; optimal cut-off = 4.3 g/dL).

The discriminative ability of hemoglobin levels at admission for predicting mortality was assessed using ROC curve analysis (Figure 6). Hemoglobin showed good predictive performance with an AUC of 0.79 (95% CI 0.68–0.89, *p* < 0.01). The optimal cut-off identified by the Youden index was 4.3 g/dL, providing a sensitivity of 57% and a specificity of 95% for mortality prediction.

Patients admitted ≥3 days after bleeding onset had a significantly higher risk of mortality compared with those admitted within ≤2 days (50% vs. 12.8%). This corresponded to a relative risk of 3.9 and an odds ratio of 6.8 (95% CI 1.9–24.9, *p* = 0.0056) (Table 9).

Mortality was strongly influenced by both admission delay and hemoglobin level (Table 10). Patients admitted within ≤2 days and with Hb ≥ 8 g/dL had excellent survival (>95%), whereas those with delayed admission (≥3 days) and severe anemia (Hb < 8 g/dL) experienced the highest mortality (>70%). Early admission appeared to attenuate the adverse impact of low hemoglobin.

Mortality was stratified by cirrhosis etiology and hemoglobin level (Table 11). In alcoholic cirrhosis, patients with Hb < 8 g/dL had markedly higher mortality (~31%) compared with those with Hb ≥ 8 g/dL (~9%). In contrast, non-alcoholic patients showed overall lower mortality, with only ~13% deaths in the low hemoglobin group and none in the high hemoglobin group.

## 4. Discussion

In this retrospective cohort of 98 patients admitted for variceal upper gastrointestinal bleeding between 2009 and 2014, we observed outcomes that are consistent with the historical burden of this condition. Despite advances in diagnosis and endoscopic therapy, VUGIB continues to carry substantial short-term mortality, in line with the reported 6-week mortality of 15–20% in international cohorts [1]. Our findings underscore the importance of early recognition, timely admission, and the adoption of evidence-based therapeutic strategies.

Our study confirms that mortality in VUGIB is driven primarily by the severity of hemorrhage at admission and by delays in hospital presentation, rather than by the underlying etiology of cirrhosis. In our cohort, alcoholic cirrhosis was predominant, in accordance with regional epidemiology and prior Eastern European studies, while viral and metabolic etiologies were less frequent. The predominance of alcoholic cirrhosis may reflect the regional patterns of alcohol consumption and the associated liver disease burden. These findings highlight the need for rapid intervention and appropriate risk stratification regardless of cirrhosis etiology.

### 4.1. Prognostic Factors and Comparison with Literature

Our study confirms that mortality in variceal upper gastrointestinal bleeding (UGIB) is determined mainly by hemorrhage severity at admission and delays in hospital presentation, rather than by cirrhosis etiology. Alcoholic cirrhosis predominated in our cohort (73%), consistent with Romanian and international data [8,9], while esophageal varices were the leading bleeding source [10]. The overall mortality of 17.3% aligns with European reports (15–22%) [11].

Hemoglobin on admission was a strong prognostic marker: patients with Hb < 5 g/dL had the poorest survival, consistent with the concept that profound anemia reflects uncontrolled bleeding and limited reserve. ROC curve analysis confirmed its discriminative value (AUC 0.79), though interpretation should consider other clinical factors [12,13]. Admission delay was also critical: both late presentation (>48–72 h) and very early admission (<6 h) were linked to higher mortality, reflecting the combined risks of uncontrolled bleeding and delayed intervention [14].

The underuse of pre-emptive TIPS in our historical cohort may have contributed to higher mortality. Prospective trials, notably García-Pagán et al., demonstrated survival benefit from early TIPS in Child–Pugh B/C patients [13], supporting current Baveno VII consensus, which emphasizes immediate vasoactive therapy, prophylactic antibiotics, and endoscopy within 12 h [15].

Taken together, our findings highlight hemoglobin levels and admission delay as key prognostic factors, reinforcing the importance of rapid referral, early endoscopy, and optimized transfusion strategies. These elements should be integrated into risk stratification and management algorithms to improve outcomes in variceal UGIB [13,14,15,16,17,18,19].

### 4.2. Etiology and Risk Stratification

In our cohort, patients admitted more than 24 h after bleeding onset had significantly worse outcomes, underscoring the importance of rapid referral and early specialized care. Previous studies consistently show that postponing admission or delaying therapeutic endoscopy increases mortality due to uncontrolled bleeding and early rebleeding [20,21,22]. Current guidelines, including ESGE and Baveno VII, recommend endoscopy within 12 h of stabilization, and our results support this timeframe [15,23].

Bleeding severity was another strong predictor: profound anemia (Hb < 8 g/dL, especially <5 g/dL) was closely linked to mortality, consistent with evidence that hemoglobin reflects both blood loss and prognosis [24,25,26,27]. Restrictive transfusion strategies (Hb 7–9 g/dL) have been shown to reduce rebleeding and death, with the pivotal trial by Villanueva et al. confirming improved survival and bleeding control [12,28,29].

Anemia severity and admission delay often coexisted, with late presenters showing greater hemodynamic instability, deeper anemia, and organ dysfunction, all contributing to poor outcomes [20,21,27]. These findings emphasize the need for efficient prehospital triage, early vasoactive therapy, restrictive but timely transfusion, and urgent endoscopic intervention.

In summary, our findings identify delayed presentation and severe anemia at admission as critical predictors of mortality in variceal bleeding, reinforcing the importance of rapid referral systems and adherence to guideline-based transfusion and endoscopic strategies [15,23,28].

### 4.3. Impact of Admission Delay and Initial Severity

Admission delay beyond 24 h was strongly associated with reduced survival, emphasizing the importance of timely referral and early intervention in variceal upper gastrointestinal bleeding (VUGIB). Prior studies have shown that postponing hospital admission or endoscopic therapy significantly increases mortality through uncontrolled hemorrhage and early rebleeding [20,21,22]. Current ESGE and Baveno VII recommendations advise endoscopy within 12 h of stabilization, and our findings support this threshold [15,23].

Bleeding severity was another major prognostic factor. Severe anemia (Hb < 8 g/dL), particularly <5 g/dL, was independently linked to mortality, consistent with evidence that low hemoglobin reflects both bleeding severity and poor prognosis [24,25,27]. Randomized trials and meta-analyses confirm that a restrictive transfusion strategy (Hb 7–9 g/dL) lowers rebleeding and mortality compared with liberal thresholds [28,29], as demonstrated in the pivotal trial by Villanueva et al. [12].

Notably, admission delay and anemia severity are interrelated, with late presenters more likely to exhibit hemodynamic instability, profound anemia, and multi-organ dysfunction, thereby amplifying risk [20,21,27]. This interplay underscores the need for coordinated prehospital triage, early vasoactive therapy, restrictive transfusion, and urgent endoscopy.

Overall, our findings confirm that delayed admission and severe anemia are key predictors of mortality in VUGIB, consistent with international guidelines [15,23,28], and highlight opportunities to improve survival through optimized referral systems and adherence to restrictive transfusion policies.

### 4.4. Therapeutic Strategies and Historical Context

During the study period (2009–2014), endoscopic therapy—mainly band ligation and, less frequently, sclerotherapy—was the cornerstone of treatment for variceal upper gastrointestinal bleeding in our center. Pharmacological therapy with vasoactive agents was inconsistently administered, and pre-emptive TIPS was rarely available. These limitations, reflecting both therapeutic standards of the time and resource constraints typical of many Eastern European centers, likely contributed to the suboptimal survival compared with contemporary series from Western Europe and North America [30,31].

Since then, therapeutic strategies have evolved substantially. Current consensus emphasizes a combined approach: immediate initiation of vasoactive therapy, routine administration of broad-spectrum antibiotics, and endoscopic band ligation within 12 h of admission [32]. Endoscopic sclerotherapy, once common, has been largely abandoned in favor of band ligation due to its superior safety profile [32].

A major advance has been the use of pre-emptive TIPS in selected high-risk patients (Child–Pugh C < 14 or Child–Pugh B > 7 with active bleeding). Randomized trials demonstrated that early TIPS reduces treatment failure and improves survival [33], although access remains limited in many low- and middle-income countries [34].

Our findings underscore the gap between available therapies during the study period and current evidence-based standards. Patients were treated mainly with conventional endoscopic procedures, while interventions now considered essential—such as early vasoactive therapy, prophylactic antibiotics, and pre-emptive TIPS—were not systematically applied. This therapeutic gap likely contributed to the higher mortality observed, reinforcing that adherence to guideline-based protocols can significantly improve prognosis in acute variceal bleeding [32,33]. It should be emphasized that our cohort reflects a historical period (2009–2014), when current standards of care such as early vasoactive therapy, routine prophylactic antibiotics, restrictive transfusion, and pre-emptive TIPS were not consistently applied. Therefore, the outcomes observed in our series should not be interpreted as representative of present-day management but rather as a benchmark of real-world practice in Eastern Europe at that time. This historical perspective also highlights the progress achieved over the last decade and underscores the survival gains expected with contemporary evidence-based strategies.

### 4.5. Strategies for the Primary Prevention of Variceal Bleeding

Primary prevention of variceal hemorrhage remains central to portal hypertension management. Current consensus recommends non-selective beta-blockers (NSBBs) as first-line therapy, with carvedilol increasingly favored due to its greater reduction in portal pressure compared with propranolol or nadolol [15]. Endoscopic variceal ligation (EVL) is an effective alternative when NSBBs are contraindicated or not tolerated [35]. Recent randomized data, including the CAVARLY trial, indicate that combining carvedilol with EVL may further reduce first bleeding events and one-year mortality [36]. For gastric varices, balloon-occluded retrograde transvenous obliteration (BRTO) shows promise, although current evidence is limited [37]. Overall, NSBBs remain the backbone of primary prophylaxis, with combination strategies considered in selected high-risk patients.

### 4.6. Secondary Prophylaxis of Variceal Hemorrhage

For secondary prevention of variceal hemorrhage, current consensus supports combining non-selective beta-blockers (NSBBs) with endoscopic variceal ligation (EVL), which is more effective than either therapy alone in reducing rebleeding [15,27,38,39]. EVL monotherapy is reserved for patients intolerant to NSBBs. Among beta-blockers, carvedilol is increasingly used due to its stronger effect on portal pressure, although propranolol and nadolol remain widely prescribed [15,35]. In gastric varices, emerging evidence suggests that adding NSBBs to endoscopic therapy may lower rebleeding risk, though survival benefit is less clear [37]. In high-risk patients or those with recurrent bleeding despite optimized prophylaxis, early trans-jugular intrahepatic portosystemic shunt (TIPS) offers an effective rescue option [27,35].

### 4.7. Endoscopic Therapy

Endoscopic variceal ligation has become the dominant endoscopic method for secondary prevention of variceal hemorrhage, progressively replacing endoscopic sclerotherapy (EIS). Evidence from meta-analyses demonstrates that EVL achieves superior variceal eradication and is associated with lower risks of rebleeding and adverse events compared with EIS [40]. Major consensus statements, including Baveno VII and the most recent AASLD guidance, recommend EVL in combination with nonselective beta-blockers (NSBBs) as the preferred strategy for secondary prophylaxis, as this dual approach lowers both rebleeding and mortality rates more effectively than either modality alone [15,41,42]. By contrast, combining EVL with sclerotherapy has not been shown to confer additional benefit and may increase complications, so it is not advised [25]. In the context of gastric varices, endoscopic cyanoacrylate injection remains the treatment of choice, with EVL considered only in selected situations [15]. Taken together, current data reinforce the role of EVL as the foundation of endoscopic management of variceal bleeding [43].

### 4.8. Efficacy and Limitations

Endoscopic variceal ligation (EVL) remains the primary endoscopic therapy for esophageal variceal hemorrhage, achieving initial hemostasis in 85–95% of cases [27,40]. Compared with sclerotherapy, EVL provides higher eradication rates, fewer recurrences, and fewer complications [40]. Because it does not address portal hypertension, recurrence is common unless combined with non-selective beta-blockers (NSBBs) [15]. Despite advances, rebleeding still occurs in 7–10% of patients within 6 weeks, and post-banding ulcer bleeding remains a relevant complication (2–10%) [44,45]. Current consensus supports EVL plus NSBBs as the most effective strategy for secondary prophylaxis, while combining EVL with sclerotherapy is discouraged due to excess adverse events [15,27,46]. For gastric varices, cyanoacrylate injection is preferred, with early trans-jugular intrahepatic portosystemic shunt (TIPS) considered in selected high-risk cases [1,27,47,48].

### 4.9. Coagulation Management and Adjunctive Therapies

Traditionally, cirrhosis was viewed as a hypo-coagulable state, with variceal hemorrhage attributed to impaired clotting factor synthesis and thrombocytopenia. More recent data support the concept of “rebalanced hemostasis,” in which decreased procoagulant activity is offset by reduced anticoagulant function and endothelial dysfunction, placing patients at risk for both bleeding and thrombosis, particularly portal vein thrombosis [30,32,42].

These insights have reshaped transfusion strategies. Fresh frozen plasma and platelet concentrates were previously given prophylactically for elevated INR or low platelet counts, but evidence shows that INR is not a reliable predictor of bleeding and that indiscriminate transfusion may increase portal pressure and rebleeding risk [30]. Current guidelines therefore recommend restricting transfusion to cases of severe thrombocytopenia (<50,000/µL) or uncontrolled hemorrhage [33,49].

Viscoelastic assays such as thrombo-elastography (TEG) and rotational thrombo-elastometry (ROTEM) provide real-time assessment of clot dynamics, offering a more accurate measure of hemostasis than conventional assays. Their use can reduce unnecessary transfusions without increasing bleeding complications in cirrhotic patients undergoing invasive procedures [34].

Adjunctive pharmacologic measures remain crucial. Prophylactic antibiotics are essential given the adverse impact of bacterial infections on outcomes [50], while proton pump inhibitors are often used peri-endoscopically to minimize post-procedural ulcer risk. Antifibrinolytics have been explored but lack sufficient evidence for routine use.

In summary, coagulation management in cirrhosis should balance bleeding and thrombosis risk through restrictive transfusion policies, integration of viscoelastic testing, and targeted adjunctive therapies as part of evidence-based variceal bleeding care.

### 4.10. Clinical Implications and Proposal for an Updated Algorithm

The findings of our study have important clinical implications. Despite therapeutic advances, outcomes in variceal upper gastrointestinal bleeding (VUGIB) remain largely determined by admission delay and bleeding severity. This underscores the need for streamlined pathways that enable early recognition, rapid referral, and strict adherence to evidence-based protocols.

Recent consensus statements, including ESGE (2022), Baveno VII (2022), and AEEH (2025), provide standardized frameworks for acute variceal bleeding management. Integrating these recommendations with our observations, we propose a revised diagnostic and therapeutic algorithm that reflects contemporary standards while addressing gaps identified in routine practice [15,51]:

1. Early triage and resuscitation with restrictive transfusion—Patients should be rapidly identified at first contact and transferred to specialized centers whenever possible. Hemodynamic stabilization must be prioritized, with a restrictive transfusion strategy (target Hb 7–9 g/dL), as liberal transfusion has been associated with increased portal pressure and higher risk of rebleeding [15,23].
2. Prompt initiation of vasoactive drugs and antibiotics—Vasoactive agents (terlipressin, somatostatin, or octreotide) should be started immediately upon suspicion of variceal bleeding, before endoscopic confirmation, and continued for 2–5 days. In parallel, broad-spectrum antibiotics are mandatory to reduce the incidence of bacterial infections, which can precipitate rebleeding and worsen survival [42,51].
3. Early endoscopy within 12 h—Endoscopic band ligation is the first-line treatment for esophageal varices and should be performed within 12 h of admission, once the patient is stabilized. Sclerotherapy may be considered only if band ligation is not feasible. For gastric varices, cyanoacrylate injection is recommended [42,51,52,53].
4. Pre-emptive TIPS in high-risk patients—In patients with advanced liver disease (Child–Pugh C < 14 points or Child–Pugh B > 7 with active bleeding), early TIPS placement within 72 h has been shown to reduce treatment failure and improve survival [42,53]. The adoption of this strategy represents a paradigm shift compared to the period covered by our study, when TIPS was rarely available.
5. Secondary prophylaxis with non-selective beta-blockers (NSBBs) plus endoscopic surveillance—To prevent recurrent bleeding, combination therapy with NSBBs (e.g., propranolol or carvedilol) and repeated band ligation is recommended. This approach reduces both rebleeding rates and overall mortality [12,42].

**Figure 7:**
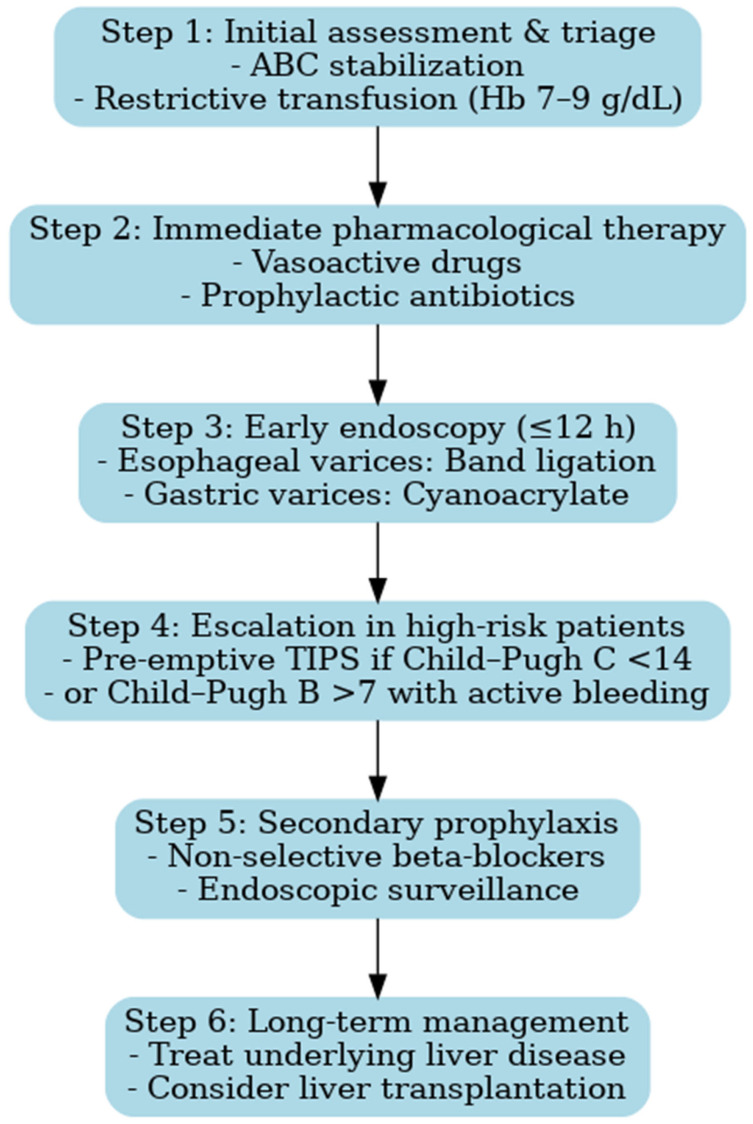
Proposed management algorithm for variceal upper gastrointestinal bleeding, integrating current guideline recommendations. Legend: ABC = Airway, Breathing, Circulation; TIPS = Transjugular Intrahepatic Portosystemic Shunt.

The implementation of such an algorithm is expected to improve both short- and long-term outcomes, particularly in healthcare settings where gaps between guideline recommendations and real-world practice remain (Figure 7). The diagnostic and therapeutic algorithm proposed here does not aim to introduce novel concepts beyond established international guidelines. Its main value lies in contextualizing these recommendations for Eastern European healthcare systems, where resources may be limited and access to advanced interventions such as pre-emptive TIPS remains restricted. By integrating evidence-based strategies into a practical framework adapted to local realities, the algorithm provides clinicians with a structured approach to patient management in similar settings. Moreover, it illustrates the progress achieved since the 2009–2014 study period and highlights the contrast between historical practices and current standards of care.

### 4.11. Strengths and Limitations

This study has notable strengths, including its status as one of the largest single-center retrospective cohorts of variceal upper gastrointestinal bleeding (VUGIB) from Eastern Europe, comprising 98 consecutive cases over a six-year period. The comprehensive collection of clinical, laboratory, and survival data enabled robust subgroup analyses, including risk stratification by etiology, hemoglobin levels at admission, and admission delay. Additionally, survival analysis using Kaplan–Meier curves provide valuable prognostic insights and establishes a historical benchmark for evaluating practice improvements. A further strength is the comparison of our findings with contemporary international guidelines, allowing for the development of an updated diagnostic and therapeutic algorithm applicable to both high-resource and resource-limited settings.

However, several limitations must be acknowledged. The retrospective design introduces potential biases related to data completeness and accuracy. As a retrospective study, it is susceptible to selection bias, reporting bias, and missing data, which can affect the reliability of the findings. Furthermore, the single-center nature of the study limits the generalizability of the findings to other healthcare systems, particularly in countries with different clinical settings and resources. As a tertiary surgical referral center, our hospital may also have received disproportionately severe cases, potentially inflating mortality rates compared with community hospitals. The single-center design, modest sample size, and retrospective methodology inherently constrain the external validity and generalizability of our findings. These limitations should be considered when interpreting the results.

Although relatively large for a single-center study in Eastern Europe, the sample size remains modest compared with multinational cohorts, limiting the statistical power of subgroup and regression analyses. The lack of standardized hemodynamic assessment (e.g., hepatic venous pressure gradient [HVPG]) precludes a precise characterization of portal hypertension severity, an increasingly important prognostic factor in cirrhosis. Additionally, systematic information on concomitant medications, including nonsteroidal anti-inflammatory drugs (NSAIDs), antiplatelet agents, beta-blockers, anticoagulants, and antiviral therapies, was not available, limiting our ability to assess their influence on bleeding risk and survival. Although comorbidities such as cardiovascular disease, diabetes, and renal dysfunction were documented, their independent prognostic impact was not evaluated. Moreover, data on alcohol abstinence and viral suppression were lacking, which limits the ability to contextualize outcomes based on etiology.

The incomplete information regarding the dose and duration of therapies, including vasoactive agents, antibiotics, and transfusions, reduces comparability with more contemporary cohorts. Importantly, during the study period (2009–2014), all patients were admitted and managed within the Surgical Department, in line with institutional protocols of that time. This organizational factor partly explains the limited use of vasoactive drugs, prophylactic antibiotics, and pre-emptive TIPS compared with current standards of care. An important limitation of our study is therefore the outdated timeframe (2009–2014). The cohort predates key advances in variceal bleeding management, including systematic use of vasoactive drugs at presentation, prophylactic antibiotics, restrictive transfusion strategies, and early pre-emptive TIPS in high-risk patients. Consequently, the direct clinical applicability of our findings to current practice is limited. However, this limitation also provides value: the data serve as a historical benchmark for Eastern Europe, allowing meaningful comparison with contemporary outcomes and illustrating the improvements achieved by guideline-based care.

Finally, the lack of long-term follow-up beyond hospitalization and six-week survival data prevented the evaluation of recurrent bleeding and long-term prognosis, which remains a critical aspect of VUGIB management. Taken together, the strengths and limitations of this study provide context for our findings and highlight the need for future multicenter prospective studies with standardized data collection, including detailed medication history, comorbidity assessment, and therapy protocols. Such studies should integrate validated risk scores (e.g., Child–Pugh, MELD), evaluate modern therapies such as early TIPS, and bridge the gap between guideline recommendations and real-world practice.

## 5. Conclusions

This study shows that variceal upper gastrointestinal bleeding (VUGIB) remains a major cause of morbidity and mortality, primarily influenced by bleeding severity at admission and delays in hospital presentation. Admission delay beyond 24 h and severe anemia are key prognostic factors. While alcoholic cirrhosis predominated in our cohort, etiology did not affect survival, highlighting the importance of timely intervention. Our findings support early vasoactive agents, restrictive transfusion, and urgent endoscopy. The underuse of pre-emptive TIPS indicates a gap in care. The proposed management algorithm aims to improve survival, and future studies are needed to refine prognostic models and optimize treatment strategies for VUGIB.

## Figures and Tables

**Table 1:** Baseline Characteristics of Patients with Variceal UGIB (n = 98).

Characteristic	Value
**Total patients**	98
**Sex**	Male 59 (60.2%), Female 39 (39.8%)
**Mean age (years)**	57.8 ± 11.7
**Residence**	Rural 73 (74.5%), Urban 25 (25.5%)
**Etiology of cirrhosis**	Alcoholic 72 (73%), Viral 18 (18%), Metabolic 8 (9%)
**Severity grade (Hb)**	Grade I: 10 (10%), Grade II: 26 (27%), Grade III: 46 (47%), Grade IV: 16 (16%)
**Mortality**	17 (17.3%)

**Table 2:** Interval between hospital admission and endoscopy (EGD) in patients with variceal UGIB.

Interval	Cases (n)	Percentage (%)
**<8 h**	4	4%
**≤48 h**	61	62%
**3–4 days**	24	24%
**>4 days**	9	9%
**Total**	98	100%

**Table 3:** Antisecretory therapy in patients with variceal UGIB (n = 98).

Therapy Type	Cases (n)	Percentage (%)
**H2 receptor inhibitors**	20	20%
**Proton pump inhibitors**	27	28%
**Combined therapy**	51	52%
**Total**	98	100%

**Table 4:** Clinical presentation in patients with variceal UGIB (n = 98).

Clinical Presentation	Cases (n)	Percentage (%)
**Hematemesis**	18	18%
**Melena**	12	12%
**Hematemesis + Melena**	68	70%
**Total**	98	100%

**Table 5:** Evolution of bleeding in patients with variceal UGIB (n = 98).

Form of Bleeding	Cases (n)	Percentage (%)
**Stopped**	60	61%
**Continuous**	20	20%
**Recurrent**	18	18%
**Total**	98	100%

**Table 6:** Multivariate logistic regression analysis of predictors of mortality in patients with variceal upper gastrointestinal bleeding (UGIB). Odds ratios (ORs) with 95% confidence intervals (CIs) and *p*-values are shown.

Variable (Reference)	Category	OR	95% CI	*p*-Value
**Interval admission delay (ref: 1–2 days)**	<6 h	22.4	1.25–399.7	**0.035**
	6–24 h	5.1	0.37–70.8	**0.220**
	3–4 days	35.3	1.59–786.1	**0.024**
	>4 days	71.0	2.16–2337.1	**0.017**
**Etiology (ref: Other)**	Alcoholic	1.4	0.35–5.4	**0.640**
**Age group (ref: 31–40 year)**	41–50 y	1.9	0.13–28.5	**0.620**
	51–60 y	0.8	0.05–13.5	**0.870**
	61–70 y	2.3	0.18–29.8	**0.520**
	>70 y	1.6	0.09–27.9	**0.740**
Variceal UGIB grade **(ref: 10–12)**	5–8	0.4	0.02–8.3	**0.550**
	8–10	0.3	0.01–6.7	**0.450**
	<5	2.8	0.15–51.8	**0.490**

**Table 7:** Univariate logistic regression analysis of mortality predictors.

Variable	OR	95% CI	*p*-Value
**Admission delay >24 h vs. ≤24 h**	0.88	0.30–2.53	**0.805**
**Admission delay ≥3 days vs. ≤2 days**	6.82	1.87–24.9	**0.004**

**Table 8:** Cross-tabulation of mortality by VUGIB grade and admission delay.

Variable	Category	Survivors n (%)	Deaths n (%)	Total
**VUGIB grade (Hb)**	<5 g/dL	5 (31.3%)	11 (68.7%)	16
	5–8 g/dL	42 (91.3%)	4 (8.7%)	46
	8–10 g/dL	25 (96.2%)	1 (3.8%)	26
	10–12 g/dL	9 (90.0%)	1 (10.0%)	10
**Admission delay**	<6 h	6 (40.0%)	9 (60.0%)	15
	6–24 h	39 (97.5%)	1 (2.5%)	40
	1–2 days	30 (96.8%)	1 (3.2%)	31
	3–4 days	4 (57.1%)	3 (42.9%)	7
	>4 days	2 (40.0%)	3 (60.0%)	5
**Total**		81 (82.7%)	17 (17.3%)	98

**Table 9:** Risk estimates for admission delay ≥3 days vs. ≤2 days.

Comparison	Risk in Exposed	Risk in Unexposed	Relative Risk (RR)	Odds Ratio (OR)	95% CI (Approx.)	*p*-Value (Fisher)
**Admission delay ≥3 days vs. ≤2 days**	50% (6/12)	12.8% (11/86)	3.91	6.82	1.9–24.9	**0.0056**

**Table 10:** Mortality stratified by admission delay and hemoglobin.

Group	Survivors n (%)	Deaths n (%)
**≤2 days + Hb ≥ 8 g/dL**	High survival (≈95%)	Very few deaths (≈5%)
**≤2 days + Hb < 8 g/dL**	Survival ~80%	Mortality ~20%
**≥3 days + Hb ≥ 8 g/dL**	Survival drops	Mortality ~40%
**≥3 days + Hb < 8 g/dL**	Very poor survival	Mortality > 70%

**Table 11:** Mortality stratified by etiology and hemoglobin.

Group	Survivors n (%)	Deaths n (%)	Total
**Non-alcoholic + Hb ≥ 8 g/dL**	100% survival	0% mortality	9
**Non-alcoholic + Hb < 8 g/dL**	~87% survival	~13% mortality	15
**Alcoholic + Hb ≥ 8 g/dL**	~91% survival	~9% mortality	23
**Alcoholic + Hb < 8 g/dL**	~69% survival	~31% mortality	32

## Data Availability

The data presented in this study are available on request from the corresponding author. The data are not publicly available due to patient confidentiality.

## Abbreviations

UGIB	Upper gastrointestinal bleeding
VUGIB	Variceal upper gastrointestinal bleeding
Hb	Hemoglobin
UGIE	Upper gastrointestinal endoscopy
ESGE	European Society of Gastrointestinal Endoscopy
TIPS	Trans-jugular intrahepatic portosystemic shunt
NSBB	Non-selective beta-blockers
EVL	Endoscopic variceal ligation
EIS	Endoscopic injection sclerotherapy
ROC	Receiver operating characteristic
AUC	Area under the curve
OR	Odds ratio
CI	Confidence interval
